# Supplementary figures and images for: Four New Species of Aspergillus Subgenus Nidulantes from China
Source: J Fungi (Basel). 2022 Nov 15;8(11):1205. doi: 10.3390/jof8111205 (PMC9697824; doi:10.3390/jof8111205)

ITS

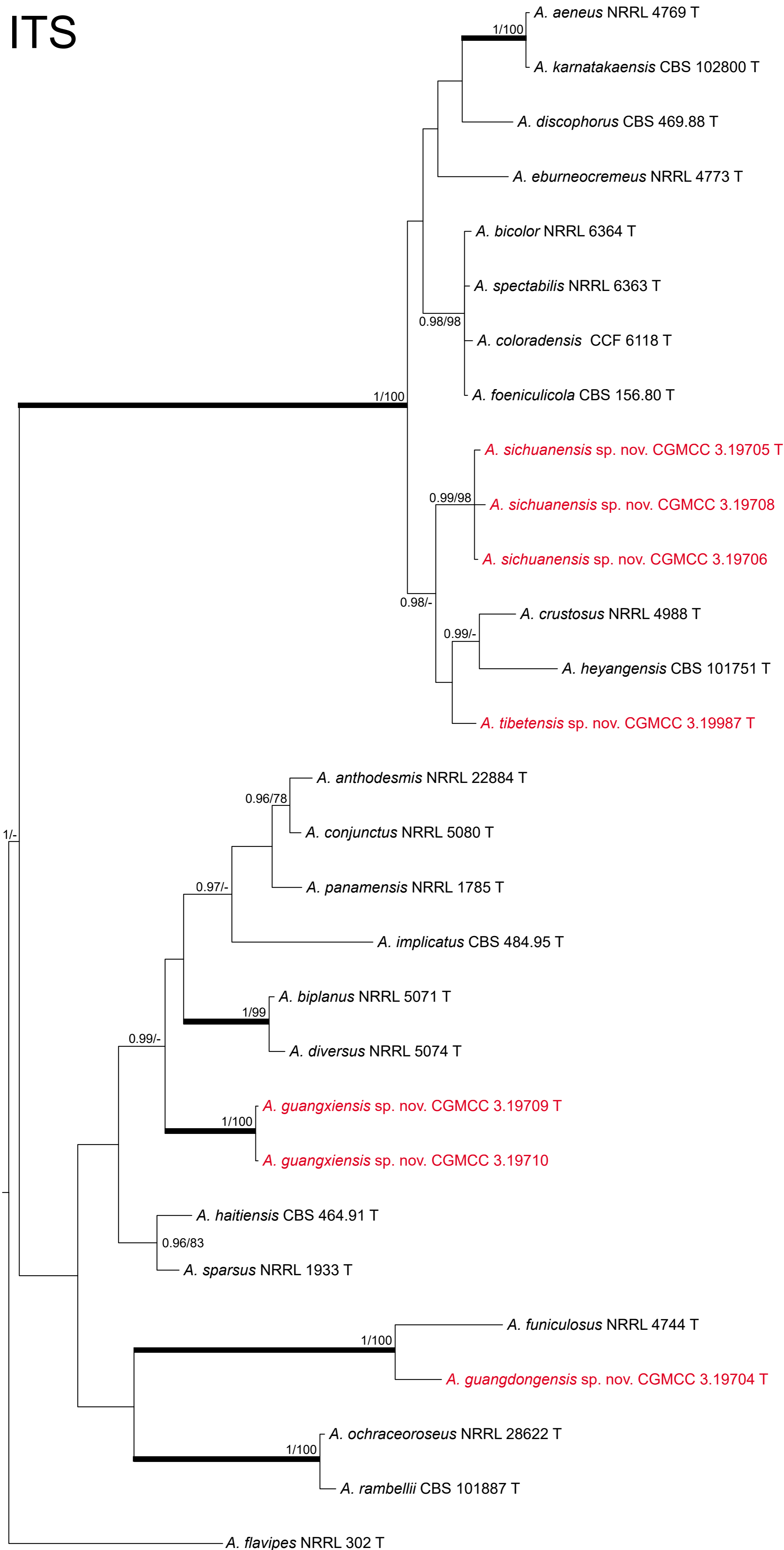

0.04

Supplement: Supplementary file 1 [file jof-08-01205-s001.zip › Figure S1.ITS.pdf]

BenA

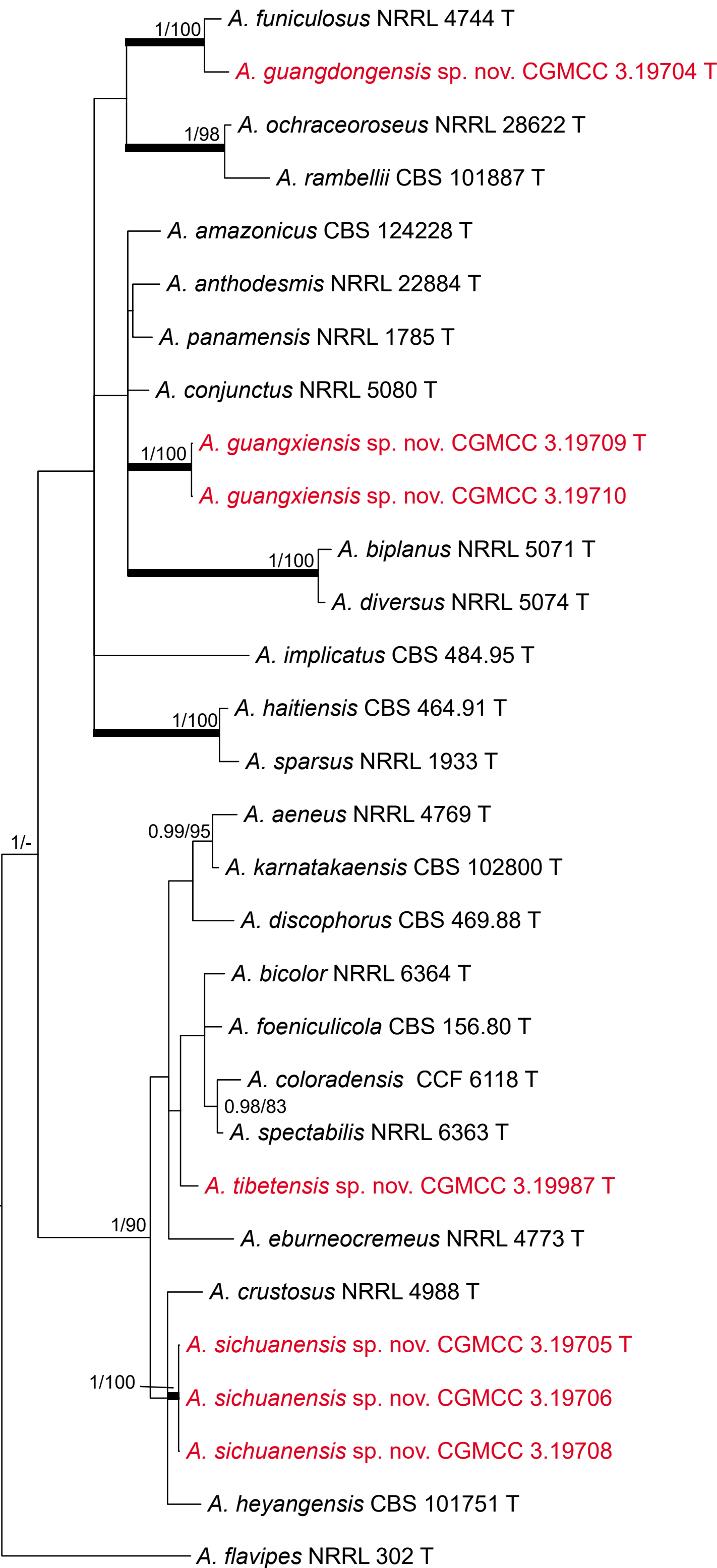

Supplement: Supplementary file 1 [file jof-08-01205-s001.zip › Figure S2.BenA.pdf]

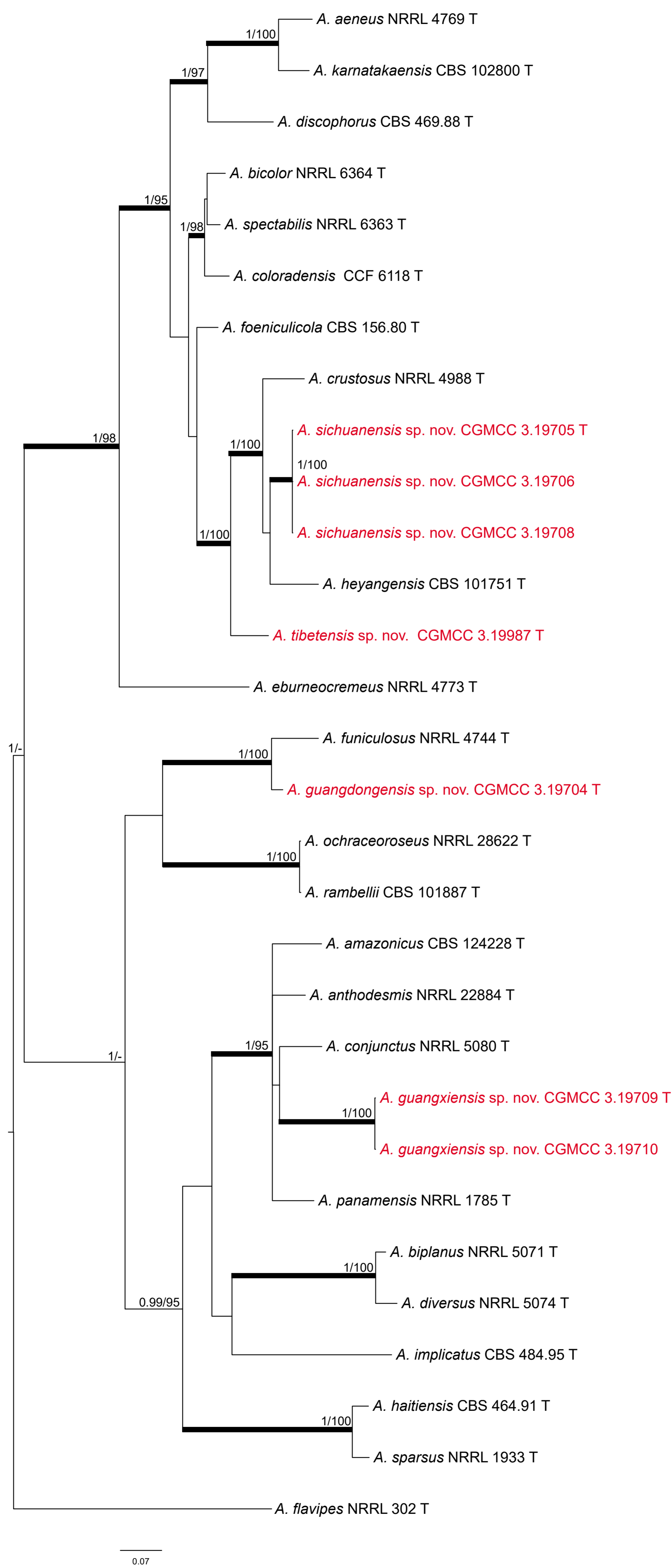

Supplement: Supplementary file 1 [file jof-08-01205-s001.zip › Figure S3.CaM.pdf]

RPB2

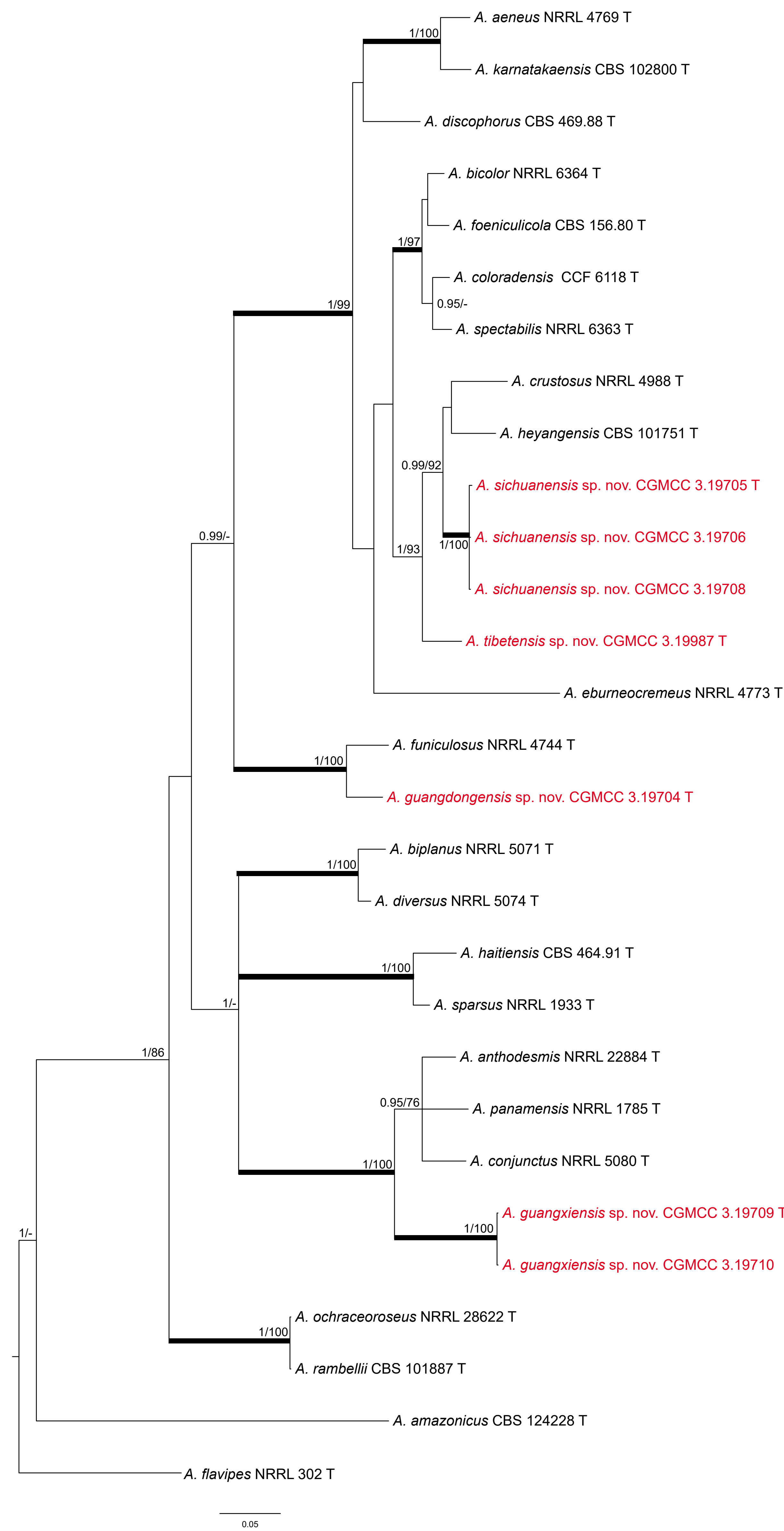

Supplement: Supplementary file 1 [file jof-08-01205-s001.zip › Figure S4.RPB2.pdf]
